# Towards More Accessible and More Inclusive PrEP to Break the Cycle of HIV in France

**DOI:** 10.1002/jia2.70118

**Published:** 2026-04-29

**Authors:** Jade Ghosn, Marie‐Laure Chaix

**Affiliations:** ^1^ AP‐HP Nord, Hôpital Bichat ‐ Claude Bernard, Department of Infectious Diseases Paris France; ^2^ Université Paris Cité, INSERM UMR 1137 IAME Paris France; ^3^ Department of Virology Hôpital Saint‐Louis, AP‐HP Paris France; ^4^ CNR VIH, Santé Publique France, INSERM UMR 1342 Université Paris Cité Paris France

**Keywords:** France, HIV prevention, pre‐exposure prophylaxis, PrEP, Treatment as Prevention (TasP)

## Abstract

**Introduction:**

Pre‐exposure prophylaxis (PrEP) represents a major advance in HIV prevention, but its rollout in France remains limited, particularly among women, migrants and socially vulnerable populations. Despite full reimbursement by the national health system, oral PrEP is still rarely prescribed outside hospital settings, hindered by organizational constraints, inaccurate medical perceptions and persistent access inequalities. In this paper, we discuss the current limits of PrEP implementation in France, identify structural and individual barriers to its uptake and highlight possible strategies to make HIV prevention more accessible for all vulnerable populations across the country.

**Discussion:**

The goal of eliminating HIV transmission by 2030 in France continues to be jeopardized by insufficient PrEP coverage. The current prevention model remains overly hospital‐centred and primarily focused on a group of men who have sex with men (MSM), which limits its broader impact. In addition to structural barriers, the insufficient diversity of prescribers and the lack of inclusive communication continue to reinforce inequalities in access. The arrival of long‐acting injectable PrEP offers an important opportunity to ensure greater discretion and better adherence. However, its success will depend on expanding the range of authorized prescribers to include gynaecologists, general practitioners and family planning clinics, supported by specific training and outreach consultations. Equally critical is strengthening public awareness campaigns and extending them beyond MSM and urban centres such as Paris, to reach diverse populations across the country. Durable improvements in PrEP uptake and retention also depend on close collaboration with community‐based organizations, building trust with marginalized populations and participatory approaches that actively listen to individuals’ concerns and lived experiences.

**Conclusions:**

France, which is lagging behind its objective of ending the HIV epidemic, has the opportunity to rethink its prevention strategy to address unmet needs and move beyond a hospital‐ and MSM‐centred model. A structural, coordinated and inclusive response is essential to expand PrEP uptake and ensure equitable protection for all populations at risk.

## 1 Introduction

Despite more than 40 years of efforts against HIV, the number of new diagnoses has remained unchanged in France [1]. Therapeutic advances and the Treatment as Prevention (TasP) strategy have led to undeniable progress for people living with HIV and their partners, substantially reducing the risk of transmission among those receiving effective treatment. However, the epidemic's trajectory has not been halted [1].

In this context, pre‐exposure prophylaxis (PrEP) represents a major advance in HIV prevention. By enabling HIV‐negative individuals to protect themselves independently of their sexual partner's HIV status, PrEP has reshaped the prevention landscape. Yet, despite its proven efficacy, uptake in France remains limited, and access is still unequal [2]. Barriers related to its method of administration, accessibility and social as well as medical perceptions surrounding PrEP hinder its full‐scale deployment [2].

In this paper, we discuss the current limits of PrEP implementation in France, identify structural and individual barriers to its uptake, and highlight possible strategies to make HIV prevention more accessible for all vulnerable populations across the country.

## 2 Discussion

### 2.1 Why PrEP Has Become Indispensable

**FIGURE 1:**
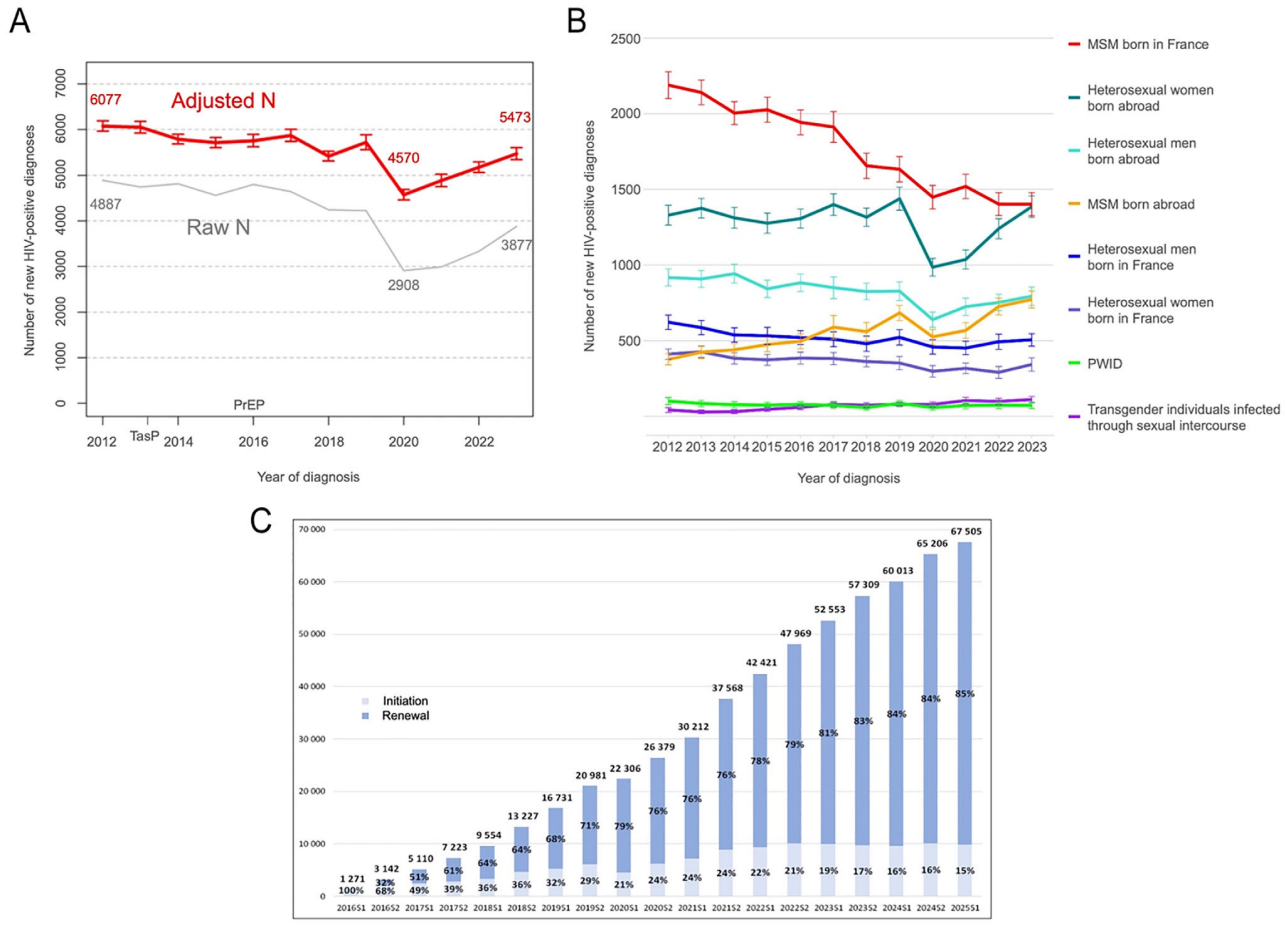
Number of new HIV diagnoses in France between 2012 and 2023 and number of PrEP users between 2016 and 2025. (A) Number of new HIV diagnoses in the overall population, data as of 30 June 2024 (raw numbers and adjusted numbers). The numbers are adjusted to account for underreporting, reporting delays and incomplete variables. (B) Number of new HIV diagnoses by gender*, probable mode of transmission and place of birth [3]. *By convention, MSM, as well as heterosexual men and women, refer here to cisgender people. (C) Number of users of PrEP initiating or renewing treatment in France, each semester between January 2016 and June 2025 [4]. Abbreviations: MSM, men who have sex with men; PrEP, pre‐exposure prophylaxis; PWID, people who inject drugs; S, semester; TasP, Treatment as Prevention.

Starting in 2013, France adopted the TasP strategy, aiming to treat all people living with HIV to achieve and maintain an undetectable viral load and thereby reduce transmission. However, between 2013 and 2016, and according to Santé Publique France, the French national public health agency, the number of new HIV diagnoses remained stable at around 6500 per year (Figure 1A), and continued to increase in some groups, notably men who have sex with men (MSM) (Figure 1B) [3].

This limited impact may partly be explained by the fact that TasP relies on early diagnosis, effective treatment and regular follow‐up, conditions that are not consistently met in practice [5]. Indeed, same‐day initiation of antiretroviral therapy is often challenging in France due to national guidelines recommending confirmation of HIV acquisition and baseline viral load before starting treatment [6]. More fundamentally, the main limitation lies in the “hidden epidemic” of undiagnosed infections, in which individuals remain unaware of their status and thus continue to contribute disproportionately to transmission. In France, more than 10,000 people are thought to be living with undiagnosed HIV [1, 7]. However, the contribution of undiagnosed individuals to onward transmission is heterogeneous across populations. Phylogenetic analyses indicate that onward transmission among heterosexual individuals typically occurs in small clusters, whereas transmission among MSM more often involves larger clusters, reflecting a higher transmission potential within MSM sexual networks [8].

Another limitation of TasP stems from the pathophysiology of HIV. Primary infection, asymptomatic in half of cases, corresponds to a period of very high contagiousness during the first weeks following seroconversion, when newly infected individuals may remain sexually active without knowing their status [9]. Indeed, up to 50% of infections among MSM originate from partners in the primary infection stage [10]. Thus, whereas TasP protects the partners of diagnosed and treated HIV‐positive individuals, PrEP offers an alternative for HIV‐negative individuals exposed to undiagnosed partners. Together, TasP and PrEP act synergistically as complementary components of a combination prevention strategy, addressing both diagnosed and undiagnosed sources of HIV transmission.

In addition to PrEP, post‐exposure prophylaxis (PEP) forms an essential component of the HIV prevention toolkit. PEP can serve as a bridge to PrEP, highlighting the importance of increasing awareness of PEP among individuals at risk of HIV.

### 2.2 Current Limits of PrEP Implementation in France

Introduced in France in 2016 and fully reimbursed for individuals at high risk of acquiring HIV, PrEP raised hopes for eradicating the virus through easy access, regular monitoring, treatment adherence and interruption of transmission chains. However, its rollout has remained largely insufficient since its launch. Two retrospective studies in France found that, in 2016 and 2020, more than 90% of individuals who recently acquired HIV would have benefited from PrEP [11, 12]. In practice, between 2016 and 2019, only about 20,000 MSM accessed PrEP in France, out of an estimated 100,000−140,000 HIV‐negative MSM who could benefit from PrEP [7, 13, 14].

Initially restricted to hospitals and sexual health centres, PrEP initiation was later extended to general practitioners in 2021. This policy change broadened prescribing capacity and enabled the cumulative number of PrEP initiations in France, across all populations, to surpass 100,000 by June 2024 [15]. However, this figure remains unsatisfactory. Indeed, modelling estimates suggest that there are 400,000 HIV‐negative sexually active MSM in France, with approximately one‐third who could benefit from PrEP [14]. Yet, only half of them are estimated to have used PrEP in the past 6 months [2]. Moreover, the profile of PrEP users has not evolved over time: they are predominantly men (96%), living in large urban areas (71%), financially comfortable (93%), with a mean age of 36 years [15, 16]. Hence, both structural and individual barriers persist, disproportionately affecting those who are younger, less urban, less educated, less affluent, less integrated into community networks, perceived as having less active sex lives and more distant from the healthcare system, or are of migrant background [2]. Indeed, the GANYMEDE study revealed that 62% of migrant MSM living with HIV acquired the infection after their arrival in France, with 13% infected within the first year [17].

Age disparities are also evident, as men under 25 years accounted for 15% of new HIV diagnoses in 2023, but represented only 20% of PrEP users, with just one‐quarter of MSM in this age group who could benefit from PrEP actually using it [2, 7, 15]. Beyond MSM, the situation of women clearly highlights the system's shortcomings. In 2023, they accounted for 32% of new HIV diagnoses, with less than 5% of PrEP initiations [7, 15]. Moreover, only 2%–4% of women at high risk of HIV acquisition in France were estimated to have initiated PrEP [18]. Migrant women are particularly underrepresented, although 60% of newly diagnosed women in France originate from sub‐Saharan Africa, and 30% acquire HIV after migration [19, 20]. It is also noteworthy that the proportion of women under 25 with a new HIV diagnosis among cisgender heterosexual women born abroad is increasing, from 14% in 2021 to 20% in 2023 [7].

Taken together, these data highlight that priority efforts for PrEP expansion in France should focus on MSM, migrants and cisgender women to support progress towards the objective of eliminating HIV transmission by 2030 [18, 21].

### 2.3 Barriers to PrEP Uptake

A primary barrier to PrEP use is its exclusive availability in oral form, which leads for some to adherence challenges and premature discontinuation. In France, between 20% and 30% of users discontinue PrEP within 6 months of initiation, particularly among those under 30 years of age and individuals experiencing socioeconomic precarity [16, 22, 23]. However, the efficacy of oral PrEP is dependent on the correct use of the prescribed dosing regimen, and missed or inconsistent intake markedly reduces its real‐world effectiveness, dropping from nearly 90% in clinical trials to approximately 60% in real‐life conditions [22]. In the United States, a retrospective cohort study involving more than 23,000 oral PrEP users found that only 40% maintained high adherence over time [24]. A quarter of participants experienced a gradual decline in adherence, another quarter discontinued almost immediately after initiation and about 10% almost never took their medication despite having a prescription [24].

French data confirm this trend. Garofoli et al. evaluated the incidence of oral PrEP discontinuation in a retrospective cohort of 2785 individuals, estimating an incidence of 10.8 per 100 person‐years [25]. Importantly, PrEP discontinuation can be appropriate during periods of reduced risk, such as a stable relationship or temporary sexual inactivity, and should be considered part of a flexible, needs‐based prevention strategy. This, however, requires health systems to enable easy and rapid PrEP re‐initiation when risk resumes, as delays in restarting may leave individuals unprotected during renewed exposure.

The oral form of PrEP also presents practical challenges, particularly regarding travel and mobility (e.g. the need to carry and remember medication while away from home) and issues of discretion (such as visible pill boxes) [26]. Furthermore, the “on‐demand” or intermittent PrEP regimen, which involves taking doses before and after sexual encounters, is currently reserved for MSM [27]. Studies have not yet demonstrated that on‐demand PrEP is effective in cisgender women. Consequently, women are required to follow a daily dosing schedule [28].

Another major barrier lies in the current prescription pathways, which are poorly adapted to the populations most in need. In France, PrEP prescribers are concentrated in CeGIDD (free centres for information, screening and diagnosis of HIV, hepatitis, and sexually transmitted infections), hospitals and general medical practices. These settings are often underutilized by individuals in precarious situations, young people, migrant women, transgender individuals, undocumented persons or those without health coverage. Limited capacity in these centres can restrict access, creating long waiting times for consultations and follow‐up. Moreover, PrEP targets individuals who self‐identify as being at risk, making perceptions, trust and patient−provider relationships critical determinants of access. Culturally responsive care, including diversity among healthcare workers, is also essential to ensure that services are approachable and relevant for all populations. The lack of integrated care hinders equitable access to PrEP and undermines the goal of an inclusive and effective prevention strategy [18].

Furthermore, in France, quick‐start or same‐day initiation of PrEP is not routinely offered, as it is required that a fourth‐generation HIV ELISA test and assessment of renal function be completed before initiating PrEP [29]. These measures, while important for safety, can delay PrEP initiation, potentially limiting timely access and coverage.

### 2.4 Strategies to Reinforce PrEP Implementation

To overcome limitations in PrEP coverage, reinforcing access requires a combination of clinical, structural and community‐based strategies.

Long‐acting injectable (LAI) PrEP offers a concrete solution to adherence difficulties. In the HPTN 083, HPTN 084, PURPOSE 1 and PURPOSE 2 clinical trials, conducted among women in sub‐Saharan Africa as well as MSM and gender‐diverse individuals, coverage with injectable PrEP (administered every 2 or 6 months) exceeded 90%, whereas adherence to daily oral PrEP varied between 10% and 75% [30, 31, 32, 33]. In these trials, injectable formulations reduced HIV incidence by 66−100 per 100 person‐years compared with oral formulations [30, 31, 32, 33]. These findings have been confirmed in real‐world studies, reporting protection rates of 99%–100% [34, 35]. LAI PrEP also improves discretion and convenience, removing the need for daily intake [36]. However, attention is needed to ensure that access to injectable PrEP does not further centralize PrEP delivery within specialized clinics, by expanding training for healthcare providers nationwide and beyond the HIV specialty. Affordability and the absence of a generic LAI PrEP in Europe remain current cost‐effectiveness considerations, highlighting the need to secure insurance coverage and avoid out‐of‐pocket expenses [37].

Strengthening PrEP implementation also requires expanding the pool of prescribers. “Outreach” consultations have demonstrated their value. For instance, sexual health consultations within family planning centres can identify prevention needs in a trusted environment, particularly for women seeking contraception or abortion care. Immediate PrEP initiation in these contexts helps remove barriers and facilitates subsequent follow‐up in CeGIDD. In parallel, it is essential to train new prescribers in key locations such as family planning centres, gynaecology departments and pharmacies, to broaden PrEP access for the most vulnerable populations [18]. Expanding prescribing to trusted healthcare providers, such as gynaecologists for women and general practitioners for migrants, can facilitate access by leveraging existing relationships of trust. In addition, providing e‐training for prescribers outside urban areas and facilitating e‐consultations for PrEP can further reduce geographic and logistical barriers. However, structural changes alone are unlikely to be sufficient.

Durable improvements in PrEP uptake and retention also depend on close collaboration with community‐based organizations, building trust with marginalized populations and participatory approaches that actively listen to individuals’ concerns and lived experiences. Co‐developing prevention strategies with community actors is essential to address stigma, misinformation, fear of discrimination and social or administrative precarity, and to ensure that PrEP delivery models are acceptable, relevant and sustainable. For example, a multidisciplinary, community‐led PrEP programme at a Paris sexual health clinic provided psychosocial, medical and legal support, coordinated with peer educators and sexual health specialists [38]. Since its implementation, the number of transgender women followed up for PrEP increased from 17 in 2016 to 129 in 2023, demonstrating the impact of community‐led, integrated care models [38].

Finally, improving access to PrEP also requires reshaping prevention campaigns. Current strategies, largely focused on MSM and urban areas like Paris, fail to resonate with other exposed populations [39]. A migrant woman, for instance, may not identify with prevention campaigns that rely on male imagery. Communication must, therefore, highlight the proven effectiveness of PrEP for women, particularly with the emergence of new modalities such as LAI PrEP, and extend beyond metropolitan hubs. Deploying inclusive campaigns tailored to the diversity of populations vulnerable to HIV is critical to ensure that PrEP is visible, relatable and accessible to all. For example, in France, the campaign “La PrEP, un geste simple contre le VIH/sida” (“PrEP, a simple step against HIV/AIDS”) has sought to normalize PrEP use and reach a broader audience, with a main focus on women of sub‐Saharan origin and men of Maghreb or Latin American origin [40].

Real‐world experiences from other countries provide valuable lessons for scaling PrEP provision in France [41, 42, 43, 44]. A successful model is exemplified by the approach used in London, at the 56 Dean Street sexual health clinic, which combines rapid HIV testing, immediate antiretroviral treatment (TasP) and widespread PrEP uptake. Between 2017 and 2024, this integrated strategy led to a 61% decline in new HIV diagnoses among clinic attendees, alongside a sharp rise in PrEP users from 170 to over 25,000, demonstrating the effectiveness of combination strategies in a concentrated prevention setting [42]. Nationwide programmes in Spain [43] and Germany [44] further illustrate that national‐scale PrEP implementation can be effective. In Spain, where PrEP became available through the public system in late 2019 and was implemented nationally in 2021, nearly 28,800 people had received PrEP by May 2024. Data from the Spanish PrEP Programme Information System show high uptake primarily among MSM, with very low HIV seroconversion rates (0.12 per 100 person‑years) [43]. Similarly, in Germany, a national PrEP monitoring system revealed an HIV incidence of 0.07 per 100 person‐years between September 2019 and August 2023, highlighting the effectiveness of structured, continuous PrEP provision combined with real‐world surveillance [44]. Collectively, these examples suggest that France could enhance the impact of PrEP by adopting coordinated, clinic‐based strategies that integrate testing, TasP and PrEP, supported by robust national monitoring systems.

## 3 Conclusions

The current HIV prevention model in France, centred on TasP and oral PrEP, has reached its limits. To reinvigorate HIV prevention, community‐led, integrated multidisciplinary care models are warranted. Integrating LAI PrEP into the national strategy may improve adherence and provide greater discretion. Expanding PrEP prescribing to gynaecologists, general practitioners and family planning clinics, supported by specific training and outreach consultations, is also crucial to reach the most at‐risk populations, who are often overlooked. Finally, inclusive prevention campaigns are essential to raise awareness and PrEP uptake. Without rapid action, France risks falling short of the UNAIDS 2030 targets.

## Author Contributions

Conceptualization of draft: JG and M‐LC. Writing – original draft: JG and M‐LC. Writing – review and editing: JG and M‐LC.

## Funding

The medical writing was funded by Kephren.

## Conflicts of Interest

JG has received honoraria from Gilead Sciences, ViiV Healthcare, GSK and Bavarian Nordic. M‐LC has no conflicts of interest to declare.

## Data Availability

Data sharing is not applicable to this article, as no datasets were generated or analysed.
